# Mouse CCDC79 (TERB1) is a meiosis-specific telomere associated protein

**DOI:** 10.1186/1471-2121-15-17

**Published:** 2014-05-22

**Authors:** Katrin Daniel, Daniel Tränkner, Lukasz Wojtasz, Hiroki Shibuya, Yoshinori Watanabe, Manfred Alsheimer, Attila Tóth

**Affiliations:** 1 Institute of Physiological Chemistry, Technische Universität Dresden, Fiedlerstr. 42, Dresden 01307, Germany; 2 Institute of Molecular and Cellular Biosciences, University of Tokyo, Yayoi 1-1-1, Tokyo 113-0032, Japan; 3 Department of Cell and Developmental Biology, Biocenter, University of Würzburg, AmHubland, Würzburg 97074, Germany

**Keywords:** Meiosis, Telomeres, Telomere attachment, CCDC79, TERB1, SUN1, Nuclear envelope, Recombination, Homologue pairing, Meiotic cohesion, SMC1B

## Abstract

**Background:**

Telomeres have crucial meiosis-specific roles in the orderly reduction of chromosome numbers and in ensuring the integrity of the genome during meiosis. One such role is the attachment of telomeres to trans-nuclear envelope protein complexes that connect telomeres to motor proteins in the cytoplasm. These trans-nuclear envelope connections between telomeres and cytoplasmic motor proteins permit the active movement of telomeres and chromosomes during the first meiotic prophase. Movements of chromosomes/telomeres facilitate the meiotic recombination process, and allow high fidelity pairing of homologous chromosomes. Pairing of homologous chromosomes is a prerequisite for their correct segregation during the first meiotic division. Although inner-nuclear envelope proteins, such as SUN1 and potentially SUN2, are known to bind and recruit meiotic telomeres, these proteins are not meiosis-specific, therefore cannot solely account for telomere-nuclear envelope attachment and/or for other meiosis-specific characteristics of telomeres in mammals.

**Results:**

We identify CCDC79, alternatively named TERB1, as a meiosis-specific protein that localizes to telomeres from leptotene to diplotene stages of the first meiotic prophase. CCDC79 and SUN1 associate with telomeres almost concurrently at the onset of prophase, indicating a possible role for CCDC79 in telomere-nuclear envelope interactions and/or telomere movements. Consistent with this scenario, CCDC79 is missing from most telomeres that fail to connect to SUN1 protein in spermatocytes lacking the meiosis-specific cohesin SMC1B. SMC1B-deficient spermatocytes display both reduced efficiency in telomere-nuclear envelope attachment and reduced stability of telomeres specifically during meiotic prophase. Importantly, CCDC79 associates with telomeres in SUN1-deficient spermatocytes, which strongly indicates that localization of CCDC79 to telomeres does not require telomere-nuclear envelope attachment.

**Conclusion:**

CCDC79 is a meiosis-specific telomere associated protein. Based on our findings we propose that CCDC79 plays a role in meiosis-specific telomere functions. In particular, we favour the possibility that CCDC79 is involved in telomere-nuclear envelope attachment and/or the stabilization of meiotic telomeres. These conclusions are consistent with the findings of an independently initiated study that analysed CCDC79/TERB1 functions.

## Background

The production of haploid gametes from diploid germ cells during meiosis is fundamental for sexual reproduction. Haploid gametes are produced by a single round of premeiotic DNA replication followed by two rounds of chromosome segregation. Ploidy reduction depends on features of chromosome behaviour that are specific to the first meiotic division [1]. One key feature is the pairing of homologous chromosomes (homologues) during the first meiotic prophase. High fidelity pairing of homologues requires chromosome movements, which are driven by the active movement of telomeres along the nuclear envelope (NE) in diverse taxa [2-5]. At the onset of meiotic prophase, telomeres associate with protein complexes that span the NE, and provide physical linkage between telomeres and cytoplasmic motor proteins. Hence, attachment of telomeres to the NE enables cytoplasmic motors to move telomeres along the NE [3,6,7].

Trans-NE protein complexes that connect telomeres to cytoplasmic motors contain SUN- and KASH-domain proteins, which are imbedded in the inner and the outer NE membrane, respectively [5]. In various yeast species, telomeres are tethered to the SUN-domain inner NE protein through meiosis-specific protein complexes [5,8,9]. In fission yeast, the linkage of telomeres to the SUN-domain (Sad1) protein requires at least two meiosis-specific connector proteins, Bqt1 and 2, whose interaction with telomeres depends on the telomeric DNA repeat-binding protein Taz1 and spRap1 [8,10,11]. Although RAP1 is a constitutive component of mammalian telomeres and is the predicted mammalian orthologue of spRap1, it is not required for telomere-NE interaction in mice [12], Nevertheless, SUN-domain proteins, SUN1 [13] and possibly SUN2 [14,15], are required for tethering telomeres to the NE during meiosis in mice. These two proteins are present in the inner NE both in somatic and meiotic cells. Therefore, meiosis-specific modifications to constitutive telomere proteins, or additional meiosis-specific telomere components, must exist in order to establish telomere-NE attachments in mammalian meiocytes.

In addition to telomere-NE attachment, protection of chromosome ends during meiotic prophase requires meiosis-specific changes in telomere biology [16]. The shelterin complex/telosome is employed in both somatic and meiotic cells to safeguard chromosome ends from DNA-damage response and enzymatic attacks, and to ensure maintenance of telomere length [17-19]. Although somatic and meiotic telomeres share known shelterin components, e.g. TRF1, TRF2 and RAP1, maintaining the stability of telomeres during meiosis is known to require an additional factor, possibly due to meiosis specific-features of recombination. The meiosis-specific cohesin SMC1B is required for both telomere stability and efficient telomere-NE attachment during the first meiotic prophase [20]. However, the function of SMC1B in telomere biology is not well understood, and the molecular nature of meiosis-specific telomere modifications remains largely unexplored.

Here we report, in line with a recently published study [21], that the coiled-coil-domain containing protein 79 (CCDC79) is a meiosis specific telomere-associated protein in mice. By investigating the behaviour of CCDC79 in wild type (wt), SUN1-deficient and SMC1B-deficient meiocytes, we identify CCDC79 as a candidate for mediating meiotic telomere-NE interaction and for stabilising meiotic telomeres in mammals.

## Results

### Expression of *Ccdc79* is restricted to male and female meiotic germ cells

To identify uncharacterised proteins that are possibly involved in meiotic chromosome biology, we screened for mouse genes whose expression is upregulated in the developing gonads upon entry of germ cells into the first meiotic prophase in both sexes (our unpublished results). *Ccdc79* was one of the identified genes.

**Figure 1:**
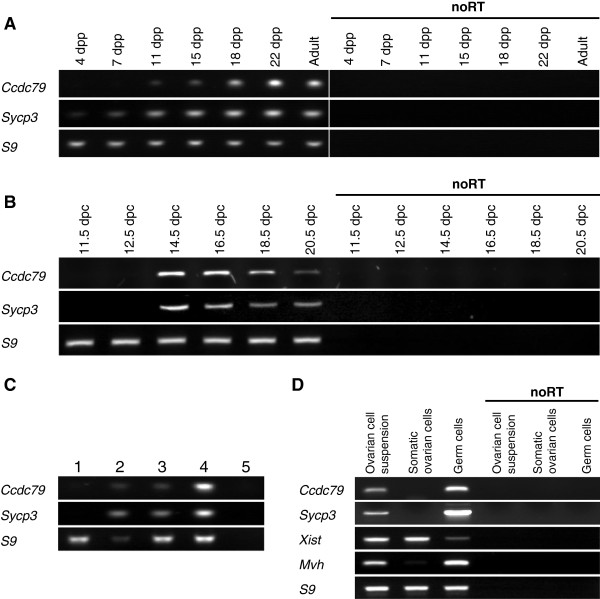
***Ccdc79 *is preferentially expressed during meiotic prophase in mice.** RT-PCR was used to measure expression of *Ccdc79*, *S9* (a “house-keeping” gene), *Sycp3* (a meiosis marker), *Mvh* (a germ cell marker), and *Xist* (a somatic cell marker in females) **(A)** RT-PCR analysis of testes collected at the indicated ages. Expression of *Ccdc79* is noticeably up-regulated as the first wave of germ cells enters meiosis after 7 days post-partum (dpp). **(B)** RT-PCR analysis of ovaries collected from foetuses at the indicated times post fertilization. Expression of *Ccdc79* is strongly up-regulated as female germ cells enter meiosis between 12.5 and 14.5 days post coitum (dpc). **(C)** RT-PCR analysis of testis and somatic tissues. cDNA was prepared from four RNA mixtures that contained RNA from adult testis and from somatic tissues in different ratios. (1) Somatic tissue mix: 1 μg of total RNA made up by mixing 59 ng of total RNA from 17 somatic tissues (see Material and Methods). (2) Testis: 59 ng total RNA from adult testis. (3) Somatic + testis mix: 1 μg of total RNA made up by mixing 59 ng total RNA from testis with 941 ng of somatic tissue mix. (4) Somatic + 5x testis: 1 μg of total RNA made up by mixing 295 ng total RNA from testis with 705 ng of somatic tissue mix. (5) noRT control with somatic + testis mix. *Ccdc79* specific PCR-products could be amplified only from templates that contained testis cDNA. **(D)** RT-PCR analysis of mixed ovarian cells, ovarian somatic cells and germ cells that were separated by fluorescence-activated cell sorting from ovaries of 16.5 dpc foetuses (see Materials and Methods). Purity of cell populations was assessed by RT-PCR specific for *Xist*, *Sycp3*, and *Mvh* marker genes. *Ccdc79* expression is restricted to germ cells in the ovary.

We examined the expression pattern of *Ccdc79* in detail in postnatal testis at different developmental stages by RT-PCR (Figure 1A). In mouse testis, the first wave of meiotic entry in germ cells occurs at 8–10 days post-partum (dpp), however the germ cell population is maintained mitotically and produces cells that initiate meiosis throughout the life of males. Expression of *Ccdc79* was not detected in testes at 4 and 7 dpp, where germ cells have not yet entered meiosis. *Ccdc79* expression became apparent at 11 dpp, coinciding with the onset of meiosis in male gonads. Once meiosis was initiated, *Ccdc79* expression level increased until 22 dpp, and remained high in adult testis. Unlike in males, germ cells enter meiosis only once during foetal development in the female gonads. Meiotic prophase is initiated in ovaries around 13.5-14.5 days post coitum (dpc). Thereafter, oocytes progress through stages of meiotic prophase relatively synchronously. Consequently, ovaries are highly enriched for germ cells of a distinct sub-stage of the first meiotic prophase at any one stage of foetal ovary development. Expression of *Ccdc79* was not detected in ovaries at 11.5 or 12.5 dpc, but we found strong expression of *Ccdc79* in 14.5 dpc ovaries (Figure 1B), where the majority of oocytes have entered the first meiotic prophase. The expression of *Ccdc79* gradually declined in later developmental stages, as oocytes progressed through prophase to the dictyate/G2 stage.

Having established that *Ccdc79* is upregulated in the gonads at the time of the first meiotic prophase, we tested if *Ccdc79* was expressed in somatic tissues. We compared *Ccdc79* expression in the testis to 17 somatic tissues by RT PCR (Figure 1C). Preferential expression of *Ccdc79* was detected in the testis of adult mice, indicating that *Ccdc79* is largely restricted to tissues that contain meiotic cells. We then asked if *Ccdc79* is expressed specifically in meiotic germ cells. Using fluorescence-activated cell sorting to separate somatic cell populations and prophase stage oocytes from foetal ovaries [22], we measured *Ccdc79* expression in the sorted cell populations by RT-PCR (Figure 1D). *Ccdc79* expression was detected in oocytes and was not detected in somatic ovarian cells, indicating that *Ccdc79* expression is restricted to meiotic cell types.

### CCDC79 shares similarities with sequence specific DNA-binding proteins

**Figure 2:**
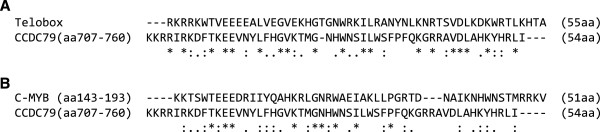
**CCDC79 is related to sequence specific DNA-binding proteins.** Amino acid sequences of the annotated Myb-like domain of *Mus musculus* CCDC79 (uniprot accession number Q8C0V1) was aligned with **(A)** the Telobox domain consensus sequence of telomere binding proteins [30] and **(B)** the annotated DNA binding Myb-domain 3 of *Mus musculus* C-MYB (uniprot accession number P06876). The indicated amino acid residues of the full-length protein sequences were compared. Identical residues in both sequences (“ * ”), conserved substitutions (“ : ”) and semi-conserved substitutions (“ . ”) are specified below the alignments.

We cloned the open reading frame of *Ccdc79* from adult testis cDNA, and reconfirmed its predicted length of 2304 bp. *Ccdc79* encodes for a protein of 768 aa, which is highly conserved in vertebrates, with an identity of 72% between the sequence of murine CCDC79 and its human homologue. CCDC79 is characterized by an N-terminal armadillo repeat and a C-terminal SANT/Myb-like domain. Armadillo repeat domains are known to mediate protein-protein interactions in a wide range of proteins with diverse functions (NCBI Conserved Domains Database accession cl02500). Myb-like domains of the SANT Superfamily [23] (NCBI Conserved Domains Database accession cl17250) are helix-turn-helix protein regions, which allow sequence specific interactions of proteins with DNA. A prominent example is the DNA-binding domain of the proto-oncogene C-MYB [24], to which members of the protein family are structurally related. Myb-like protein domains occur mainly as multiple repeats in proteins with diverse functions. However, Myb-like domains, in particular a single copy of the domain in the C-terminal, have emerged as being characteristic for proteins interacting with telomeric DNA sequences in a wide range of species e.g. in yeast, plants and mammals [25-28]. A consensus sequence, called the telobox, has been determined for such telomere-binding Myb-like domains in comparative studies, which revealed that the telobox can be distinguished from Myb-like domains of other sequence specific DNA-binding proteins [29,30]. Alignment of CCDC79 with the DNA-interacting domain of C-MYB and with the telobox revealed that the Myb-like domain of CCDC79 is more similar to the telobox (19 identical out of 54 amino acids, Figure 2) than to the DNA-interacting Myb-like domain of C-MYB (9 identical out of 54 amino acids, Figure 2). Based on this observation and the C-terminal position of the Myb-like domain in CCDC79, we hypothesised a role for the protein in meiotic telomere biology.

### CCDC79 associates with telomeres during the first meiotic prophase

**Figure 3:**
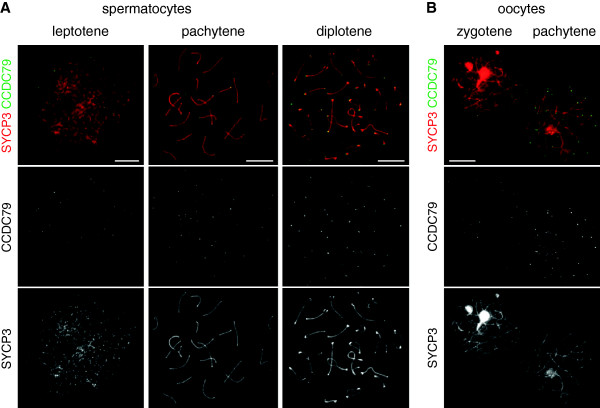
**CCDC79 localizes to the ends of chromosome axes during the first meiotic prophase.** CCDC79 and SYCP3 were detected by IF on nuclear surface spreads of **(A)** spermatocytes and **(B)** oocytes that were obtained from adult testis or 15.5 dpc ovaries, respectively. Stages of imaged meiocytes are indicated. Scale bars 10 μm.

To gain insight into the possible functions of CCDC79 during meiosis we raised antisera against a C-terminal 103 aa peptide of the protein and affinity purified the anti-CCDC79 antibodies (see Material and Methods). To examine the cellular localization of CCDC79 in meiocytes we performed immunofluorescence (IF) for CCDC79 and the axial element component SYCP3 on surface spread nuclei of adult testis (Figure 3A). The pattern of anti-SYCP3 staining was used to identify spermatocytes at distinct sub-stages of meiosis prophase I. CCDC79 first appeared in leptotene as foci on the chromatin. During early-zygotene it appeared that the CCDC79 foci co-localized with the ends of forming axial elements. Indeed, accumulation of CCDC79 as foci on virtually all the chromosome ends became apparent once axial elements had formed along the entire length of chromosomes in late-zygotene and pachytene, which suggested CCDC79 localization to telomeric regions of the chromosomes. The association of CCDC79 to the ends of chromosome axes was detected until diplotene. CCDC79 staining of chromosome ends weakened as the chromosome axis disassembled, concurrent with the progression from prophase to the first meiotic metaphase (data not shown). We did not detect a distinct anti-CCDC79 signal in spermatocytes after the first meiotic division, or in nuclear surface spreads of somatic testis cells. CCDC79 localization in oocytes resembled the localization observed in spermatocytes, i.e. CCDC79 associates to chromosome ends throughout the first meiotic prophase in oocytes and disappears from telomeres after progression to the dictyate stage (Figure 3B). Antibodies against CCDC79 obtained from the sera of two different animals showed similar immunostaining patterns in IF experiments, and were validated for specificity by immunolabeling of CCDC79-deficient spermatocytes as a negative control (Additional file 1: Figure S1). GFP tagged versions of CCDC79 overexpressed in testis have been recently reported also to localize to telomeres [21], reconfirming the observed immunostaining pattern.

### Localization of CCDC79 to chromosome ends tightly correlates with establishment of telomere-NE attachment during early prophase

**Figure 4:**
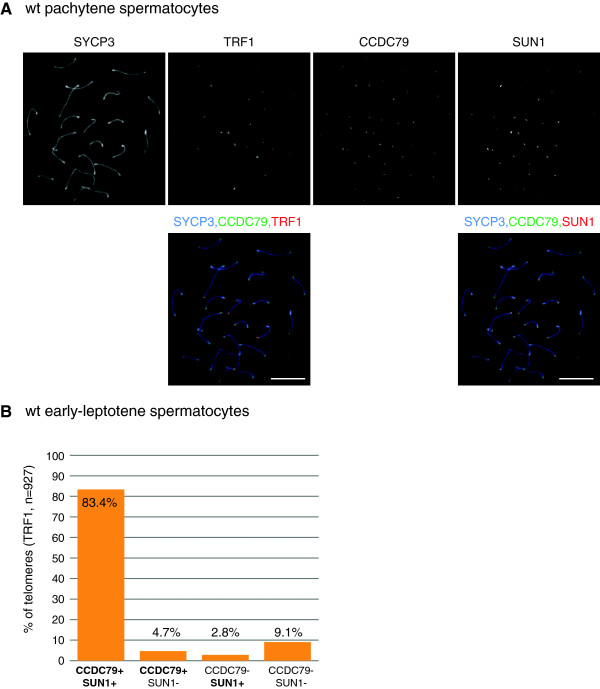
**CCDC79 and SUN1 co-localize with telomeres during the first meiotic prophase.** CCDC79, SYCP3, TRF1 and SUN1 were detected by IF on nuclear surface spreads of spermatocytes obtained from 15 dpp testis. **A)** CCDC79 co-localizes with both TRF1 and SUN1, as shown in the two overlay images (SYCP3-TRF1-CCDC79 and SYCP3-SUN1-CCDC79) of the same pachytene stage spermatocyte. **(B)** Quantification shows the fraction of all telomeres that are marked by only CCDC79 (CCDC79+ SUN1-), only SUN1 (CCDC79- SUN1+), both CCDC79 and SUN1 (CCDC79+ SUN1+), or neither CCDC79 nor SUN1 (CCDC79- SUN1-) in early leptotene stage spermatocytes. Telomeres were detected by TRF1 staining. Scale bars 10 μm.

At the onset of the first meiotic prophase, chromosomes ends are recruited to the NE via their interaction with specialized attachment sites. These structures contain the inner nuclear membrane protein SUN1. Due to the proximity of telomeres and SUN1 in the telomere attachment plates, light microscopy cannot resolve immunofluorescence detected signals of SUN1 and telomere proteins, such as TRF1, which are therefore detected as single co-localised foci. Accordingly, we found that CCDC79 foci co-localised with both TRF1 and SUN1 foci (99.4%, n = 501 telomeres) at the ends of nearly all chromosome axes in pachytene spermatocytes that were co-immunolabeled by anti-CCDC79, anti-TRF1 and anti-SUN1 antibodies (Figure 4A).

Given our finding that CCDC79 foci first appeared in leptotene, it was possible that CCDC79 association with telomeres coincided with the attachment of telomeres to the NE and with the formation of telomere attachment plates. To test this possibility we detected CCDC79, TRF1 and SUN1 in surface spreads of early-leptotene spermatocytes and quantified co-localization between the foci of these proteins (Figure 4B). We found that the vast majority (83.4%) of TRF1 foci, which constitutively marks telomeres, was associated with both anti-CCDC79 and anti-SUN1 signal. The second largest fraction (9.1%) of TRF1 foci co-localized with neither CCDC79 nor SUN1, and only very small fractions of foci showed co-localization with either CCDC79 (4.7%) or SUN1 (2.8%) foci. Thus, CCDC79 tends to associate with telomeres that also bind to SUN1 in both leptotene and pachytene, and CCDC79 is missing from the majority of telomeres that lack SUN1 in the early leptotene stage. This indicates that CCDC79 association with telomeres largely coincides with the initiation of telomere-NE attachment in wt spermatocytes.

### Telomeres that fail to attach to the NE tend to lack CCDC79 in SMC1B-deficient spermatocytes

**Figure 5:**
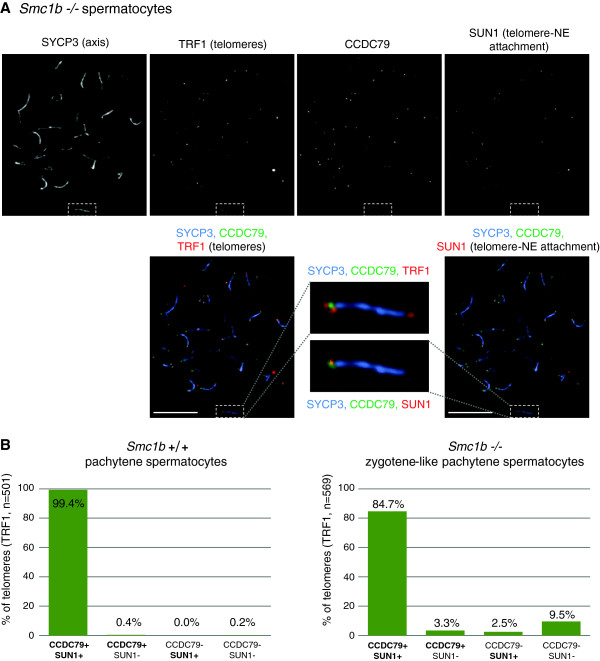
**Telomeres not bound by SUN1 tend to lack CCDC79 in *Smc1b -/- *spermatocytes.** Chromosome axes are shorter, and homologue alignment and SC formation are impaired in *Smc1b -/-* spermatocytes as compared to wt spermatocytes (see Figure 4). Therefore, the stage that is equivalent to wt pachytene is called the zygotene-like pachytene stage in the *Smc1b -/-* mutant. SYCP3 (chromosome axes), TRF1 (telomeres), SUN1 (telomere attachment plate) and CCDC79 were detected by IF in nuclear surface spreads of wild type (*SMC1b +/+,* not shown, see Figure 4) and SMC1B-deficient (*Smc1b -/-*) spermatocytes obtained from adult testis. **(A)** Images of an *Smc1b -/-* spermatocyte at a zygotene-like pachytene stage are presented: upper panel shows single channel images of the four detected proteins, lower panel shows the overlay of SYCP3/axis with either CCDC79 and TRF1 signal (left) or CCDC79 and SUN1 signal (right). Enlarged views of the same individual chromosome (dashed line box) from both merged images are indicated. On the highlighted chromosome, CCDC79 is absent from one of the TRF1 marked telomeres (right, upper panel). The same chromosome end also lacks SUN1 (right, lower panel). Scale bars 10 μm. **B)** Quantification shows the fraction of telomeres, at which CCDC79 and/or SUN1 were detected in *Smc1b +/+p*achytene spermatocytes and *Smc1b -/-* zygotene-like pachytene spermatocytes. Telomeres were detected by TRF1 staining. CCDC79 and SUN1 co-localize on almost all telomeres (99.4%) in wt spermatocytes, and only on 83.7% of all telomeres in SMC1B-deficient spermatocytes. The mutant spermatocytes exhibit a much higher rate of CCDC79-free telomeres (12%) as compared to wt spermatocytes (0.2%). The large majority (76% = 9.5% out of 12%) of these CCDC79-free telomeres also fail to associate with SUN1.

The observation that CCDC79 was preferentially associated with telomeres that established connection to the NE in early prophase wt spermatocytes indicated that CCDC79 might be a constituent of protein complexes that connect telomeres to the cytoplasmic cytoskeleton through the NE. To test this hypothesis, we addressed if CCDC79 localisation to telomeres correlated with telomere attachment to the NE in a genetic background where telomere association with the NE was partially disrupted due to a deficiency in SMC1B (Figure 5). SMC1B is a meiosis specific sub-unit of the cohesin complex which is required for correct chromosome axes length, orderly synaptonemal complex formation between homologous chromosomes, telomere stability, and efficient telomere-NE attachment during meiotic prophase [31]. A subset of telomeres fails to attach to the NE, as evidenced by the absence of SUN1 from such telomeres in SMC1B-deficient spermatocytes. To address if CCDC79 localized to telomeres that failed to attach to NE, we detected CCDC79, TRF1, SUN1 and SYCP3 in SMC1B-deficient spermatocytes by immunolabeling (Figure 5A). We found that the vast majority (96%) of telomeres that bound to CCDC79 also bound to SUN1 (Figure 5B, 88% of all telomeres were positive for CCDC79, and 96% of these, or 84.7% of all telomeres, were positive for both CCDC79 and SUN1). In addition, most telomeres (79%) that lacked CCDC79 failed to bind SUN1 in *Smc1b-/-* spermatocytes (Figure 5B, 12% of all telomeres were negative for CCDC79, and 79% of these, or 9.5% of all telomeres, were negative for both CCDC79 and SUN1, n = 569 telomeres).

Thus, there is a strong correlation between the localization of CCDC79 to telomeres and the attachment of telomeres to the NE in both wt and *Smc1b-/-* spermatocytes, consistent with the possibility that CCDC79 constitutes part of the protein complex that forms bridges between chromosome ends and the cytoplasmic cytoskeleton during meiotic prophase.

### Recruitment of CCDC79 to telomeres is independent of meiotic telomere attachment

**Figure 6:**
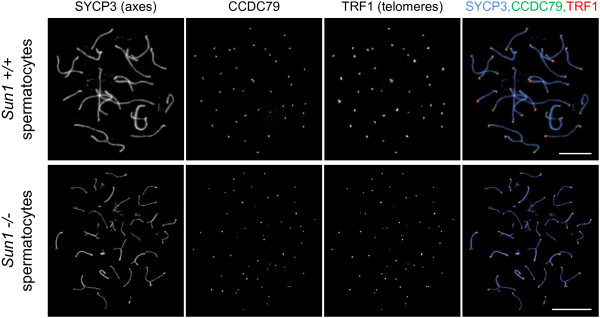
**CCDC79 localization to telomeres does not require SUN1 and telomere attachment to the nuclear envelope.** TRF1, CCDC79 and SYCP3 were detected by IF on nuclear surface spreads of spermatocytes obtained from adult wt (*Sun1 +/+)* or SUN1-deficient mice (*Sun1 -/-*). CCDC79 associates with telomeres, despite impaired telomere attachment to the nuclear envelope in the absence of SUN1. Scale bars 10 μm.

The strong correlation between the telomere-NE attachment and the localization of CCDC79 to meiotic telomeres raises the possibility that CCDC79 localization is a prerequisite for telomere-NE attachment, or that telomere-NE attachment is a prerequisite for CCDC79 localization. To test the latter, we examined the localization of CCDC79 in *Sun1 -/-* spermatocytes (Figure 6), in which the telomere-NE attachment is severely impaired. We observed wt–like localization of CCDC79 foci to chromosome ends (CCDC79 is detected at 97.4% of telomeres, n = 537) in the absence of SUN1. Thus, CCDC79 is able to interact with telomeric structures independent of telomere attachment to the NE.

## Discussion

Telomeres perform meiosis-specific functions that are essential for ploidy reduction and maintenance of genome integrity during meiosis. To facilitate active chromosome movements and ensure high fidelity pairing of homologues during early meiotic prophase, telomeres must attach to trans-NE protein complexes, thereby establishing a link to cytoplasmic motor proteins [3,16]. DNA double strand breaks are introduced into the genome and are required for homologue pairing and formation of inter-homologue crossovers. The breaks are subsequently repaired by recombination machinery that is substantially altered during meiosis, in comparison to mitosis [1]. Chromosomal DNA ends resemble DNA double strand breaks, and one of the main roles of telomeres is to protect chromosome ends and prevent undesired recombination events. It is therefore expected that meiotic telomeres need to adapt to meiosis-specific characteristics of recombination [16]. Consistent with this notion, maintenance of the structural integrity of telomeres during the first meiotic prophase requires the meiosis-specific cohesin SMC1B [20]. Despite the importance of these meiosis-specific features of telomere biology, the molecular changes in telomeres that underpin telomere-NE attachment and maintenance of telomere integrity during meiosis have remained largely unexplored.

We identified CCDC79 as a protein that associates with telomeres specifically during the first meiotic prophase in mice. We found that CCDC79 and SUN1 association with telomeres tightly correlated with each other in both wt and SMC1B-deficient meiocytes. This indicates that CCDC79 association with telomeres coincides with telomere attachment to the SUN1 containing trans-NE protein complexes.

We further tested the relationship between CCDC79 and telomere-NE attachment by examining CCDC79 localization in the *Sun1-/-* spermatocytes, where telomeres attachment to the NE is severely reduced. The observation that CCDC79 localization to chromosome ends is unaffected in SUN1-deficient spermatocytes shows that CCDC79 localization to telomeres does not depend on SUN1 and telomere-NE attachment. This also indicates that CCDC79 is not part of the protein complex that extends from SUN1 toward the cytoplasmic motors through the NE. Rather, these observations are consistent with the possibility that CCDC79 is positioned between the telomeric DNA and SUN1 at sites of telomere-NE attachment.

In agreement with this scenario, CCDC79 contains a telobox-like Myb domain at its C terminus, a feature that is shared by shelterin complex components that bind to telomeric repeats [29,30]. Thus, the domain structure of CCDC79 indicates a potential for CCDC79 to interact directly with telomeric repeats.

Our observations indicate a role for CCDC79 in meiotic telomere biology. In particular, the behaviour of CCDC79 in SMC1B-deficient meiocytes is informative. In the absence of the meiosis-specific SMC1B cohesin, meiocytes display a diverse set of telomere phenotypes during prophase. These include a failure to attach 13-20% of telomeres to the NE ([20,31] and our observations), and structural abnormalities that affect a subset of telomeres in each individual meiocyte. Such structural abnormalities include shortening of telomeres, detachment of telomeres from the meiotic chromosome axes, telomere fusions, and the formation of stretched out telomeres [20,31]. Importantly, failure of telomeres in NE attachment is correlated with the absence of CCDC79 from telomeres (this work) and with shortened telomere length [20], and is not correlated with other structural abnormalities of telomeres in *Smc1b-/-* meiocytes [20]. Thus, our experiments identify CCDC79 as a protein that may contribute to both the stabilization of telomeres during meiosis and the formation of a “bridge” that tethers telomeres to SUN1 and the inner NE.

Consistent with these findings, an independently initiated study recently revealed key roles for CCDC79 (TERB1) in the recruitment of telomeres to the NE and in the recruitment of cohesins to telomeres to maintain the structural integrity of telomeres during meiosis [21]. Given these CCDC79 functions, our observation that efficient CCDC79 recruitment to telomeres requires the meiotic cohesin SMC1B indicates synergy and mutual dependency between CCDC79 and meiotic cohesion functions at telomeres. At this point it remains unresolved if SMC1B and/or other cohesins have a direct role in CCDC79 recruitment to telomeres or if this role is more indirect and is exerted via stabilisation of telomere length and structure. Future experiments are required to address this question, as well as to dissect the functional interaction between meiotic cohesins, CCDC79 and telomeres, and also to provide a mechanistic understanding of the behaviour and functions of meiotic telomeres in mammals.

## Conclusion

Our results suggest that the meiosis-specific protein CCDC79 and its functional interaction with meiotic cohesins are involved in the meiosis-specific behaviour of telomeres. Investigation of the molecular functions of CCDC79 will provide an opportunity to explore, at the molecular level, meiosis-specific aspects of mammalian telomere biology that have been inaccessible, despite their apparent importance in ensuring a high fidelity of meiotic recombination and the integrity of the genome in the germline.

## Methods

### Animals

For expression analysis wild type (wt) tissue was isolated from C57BL/6JOlaHsd mice. To stage embryonic development, the day of detection of a vaginal plug was marked as 0.5 days post coitum (dpc). Analysed null mutant mice strains (*Smc1β -/-*, *Sun1 -/-*), have been described previously [31,32]. Experimental animals were compared with controls from the same litter (where possible) or from other litters of the same mating. All animals were bred and maintained under pathogen-free conditions according to animal welfare regulations provided by the animal ethical committee of the Technische Universität Dresden.

### Antibody generation

Anti-CCDC79 antibodies were raised against the C-terminal 103aa of CCDC79 (ENSEMBL Accession ENSMUSP00000067324). The corresponding cDNA fragment of Ccdc79 was sub cloned into the *Escherichia coli* expression vector pDEST17 (Cat#11803012, Invitrogen) and the protein was expressed in fusion with N-terminal 6xHis-tag and purified on Ni-Sepharose (Cat#17-5318-01, Amersham, GE Healthcare). Immunisation experiments were carried out at Harlan Laboratories immunisation department, Hillcrest UK. Two Hartley guinea pigs were treated using a modified version of the standard Harlan three-month guinea pig immunisation protocol. This included injection of 175 μg denatured recombinant protein in 100 μl of wet polyacrylamide gel slices at day 0, 14, 21, 49 and 77. At day 0 a small fraction of preimmune blood was taken as a control. Sera containing polyclonal antibodies against the injected protein were taken at day 84 (production bleed) and day 91 (final bleed). Polyclonal antibodies were affinity purified on antigen coupled Sepharose Beads (Cat# 17-0906-01, Amersham, GE Healthcare).

### RNA-isolation and RT-PCR

Total RNA was isolated from fresh adult mouse testis tissue or frozen embryonic gonads using the RNeasy Mini Kit (Qiagen). Mouse total RNA samples from different mouse somatic tissues were purchased via Ambion (Cat#7800) and Zyagen (Cat#MR-010). One or half a microgram of total RNA was reverse transcribed using Superscript III (Cat#18080-044, Invitrogen) and oligo dT (20) primers. In no-RT controls the reaction mixture contained water instead of reverse transcriptase. RT-PCR was performed using the *Ccdc79-*specific primers 5′-TGTGGTCTTTCCCCTTTCAG-3′ and 5′-AGGACCGAATCTCCTCCAGT-3′ and primers for the control genes *Sycp3*, *Mvh*, *Xis*t, *S9,* whose sequences were described previously [22]. The cycling conditions were: 94°C 3 min; 94°C 30 s, 54°C 30 s, and 72°C 25 s for 30 cycles; and 72°C 7 min. The 17 mouse somatic tissues used in gene expression profiling (Figure 1A) were: liver, brain, thymus, heart, lung, spleen and kidney (acquired via Ambion) and mammary gland, pancreas, placenta, salivary gland, skeletal muscle, skin, small intestine, spinal cord, tongue and uterus (acquired via Zyagen). Fluorescence-activated cell sorting of cell populations from whole embryonic female genital ridges for gene expression analysis, RNA-isolation from these samples and associated RT-PCRs were performed as previously described [22].

### Immunofluorescence (IF)

Nuclear surface spreads of spermatocytes and oocytes were prepared as described previously [33] with slight modifications. In brief, dense cell suspensions were prepared in PBS by maceration and vigorous pipetting of the gonads. Cell suspensions were diluted 20× in 100 mM sucrose in 5 mM sodium borate buffer (pH 8.5). S-fix fixative (1% paraformaldehyde, 10 mM sodium borate buffer pH 9.2, 0.15% Triton X-100) was placed on glass slides and cell suspension was placed in small droplets on the surface of the fixative. Samples were incubated for two hours at room temperature in a humid chamber. Following fast drying under a hood, the slides were washed two times for one minute with 0.4% Agepon (AgfaPhoto) and another three times for one minute with water. Slides were used immediately or kept at 4°C in PBS pH 7.4 until IF staining. Nuclear surface spreads of *Sun1-/-* spermatocytes were prepared according to the previously published protocol [34].

Before immunostaining surface spreads, samples were blocked with blocking buffer (2% BSA (Cat# A2153, Sigma), in PBS pH 7.4) for 30 min. Primary antibodies diluted in blocking buffer were applied to samples for three hours or overnight at 37°C in a humid chamber. Slides were washed three times with PBS and incubated with secondary antibodies for 1 h, and finally mounted in Vectashield mounting medium with DAPI (Cat#H-1200, Linaris).

Primary antibodies used in this study were as follows: guinea pig anti-CCDC79_1 (1:200) and guinea pig anti-CCDC79_2 (1:200), monoclonal mouse anti-SYCP3 II52F10 (1:2, a gift from R. Jessberger) [35], rat anti-SYCP3 (Y. Watanabe), rabbit anti-SUN1 (1:1000, Cat#ab74758, Abcam), rabbit anti-TRF1 (Y. Watanabe), goat anti-TRF1 E-15 (1:500, Cat#sc-5475, Santa Cruz). Donkey secondary antibodies conjugated with DyLight405 and DyLight649 (Jackson ImmunoResearch Europe Ltd.) were used at a dilution of 1:300, donkey secondary antibodies conjugated with either Alexa Fluor 488 or 568 (Molecular Probes/Invitrogen) were used at a dilution of 1:600. Fluorescence was visualised with Zeiss Axiophot fluorescence microscope.

Staining patterns were assessed manually in 100 or more nuclear spreads, including at least 30 nuclei of each particular meiotic sub stage. Cells were initially identified and characterized based on the localization pattern of the axial element component SYCP3, before the appropriate additional wavelengths were captured to determine the pattern of the co-stained proteins. Two independent sets of nuclear spreads were examined from each mutant. Except for the *Sun1-/-m*utant, we examined nuclear spreads from at least three different animals. Images displayed in the figures are representative of the most predominant staining patterns. Presented images were processed for background correction and false coloured in overlays using Adobe Photoshop CS5. In nuclear surface spreads of spermatocytes and oocytes, where most of the detergent soluble cell material is removed, both antibodies raised against CCDC79 showed similar staining patterns.

### Quantification of foci numbers of proteins detected by IF

To assess protein behaviour, foci specific to the proteins of interest were counted and compared in randomly selected nuclei of wt and mutant meiocytes of the indicated stage. Mutant and wt spreads were stained simultaneously using the same antibody mixes. Imaging of the cells for each experiment was carried out on the same day using the same microscope and camera settings. SYCP3/axis staining was used to select spermatocytes or oocytes of distinct stages. Telomeres were identified by TRF1-staining.

### Protein alignment analysis

Myb-like domains of *Mus musculus* CCDC79 and *Mus musculus* c-MYB were determined via annotation according to the UniProt Protein knowledgebase (http://www.uniprot.org). Accession numbers of the proteins and compared amino acid ranges of the full length proteins are indicated in Figure 2. The Telobox consensus sequence was analysed as described previously [30]. Protein sequence alignments were performed by using ClustalOmega.

## Abbreviations

aa: Amino acid; bp: Base pairs; dpc: Days post coitum; dpp: Days post-partum; IF: Immunofluorescence; NE: Nuclear envelope; wt: Wild-type.

## Competing interests

The authors declare that they have no competing interests.

## Authors’ contributions

Conceived and designed the experiments: KD AT. Performed the experiments: KD DT LW. Confirmed antibody specificity in *Ccdc79/Terb1-/-* spermatocytes: HS, YW. Analyzed the data: KD AT. Contributed reagents/materials/analysis tools: MA. Wrote the paper: KD AT. All authors read and approved the final manuscript.

## Supplementary Material

Additional file 1: Figure S1 Anti-CCDC79 signal is absent on telomeres in CCDC79 (TERB1)-deficient spermatocytes. TRF1, CCDC79 (TERB1) and SYCP3 were detected by IF on nuclear surface spreads of spermatocytes obtained from the testis of adult wt (*Terb1 +/+,* upper panel) or CCD79 (TERB1)-deficient mice (*Terb1 -/-,* lower two panels). Anti-CCDC79 staining was imaged with an exposure equal to the wt control (second panel) or with an intensified exposure (third panel, see insert with magnification of three individual telomeres). No anti-CCDC79 (TERB1) staining could be detected on telomeres in *Terb1-/-*spermatocytes. Scale bars 5 μm. Click here for file
