# Supplementary material for: Mouse CCDC79 (TERB1) is a meiosis-specific telomere associated protein
Source: BMC Cell Biol. 2014 May 22;15:17. doi: 10.1186/1471-2121-15-17 (PMC4038382; doi:10.1186/1471-2121-15-17)
Supplement: Additional file 1: Figure S1 — Anti-CCDC79 signal is absent on telomeres in CCDC79 (TERB1)-deficient spermatocytes. TRF1, CCDC79 (TERB1) and SYCP3 were detected by IF on nuclear surface spreads of spermatocytes obtained from the testis of adult wt (Terb1 +/+, upper panel) or CCD79 (TERB1)-deficient mice (Terb1 -/-, lower two panels). Anti-CCDC79 staining was imaged with an exposure equal to the wt control (second panel) or with an intensified exposure (third panel, see insert with magnification of three individual telomeres). No anti-CCDC79 (TERB1) staining could be detected on telomeres in Terb1-/-spermatocytes. Scale bars 5 μm. [file 1471-2121-15-17-S1.pptx]

## Slide 1
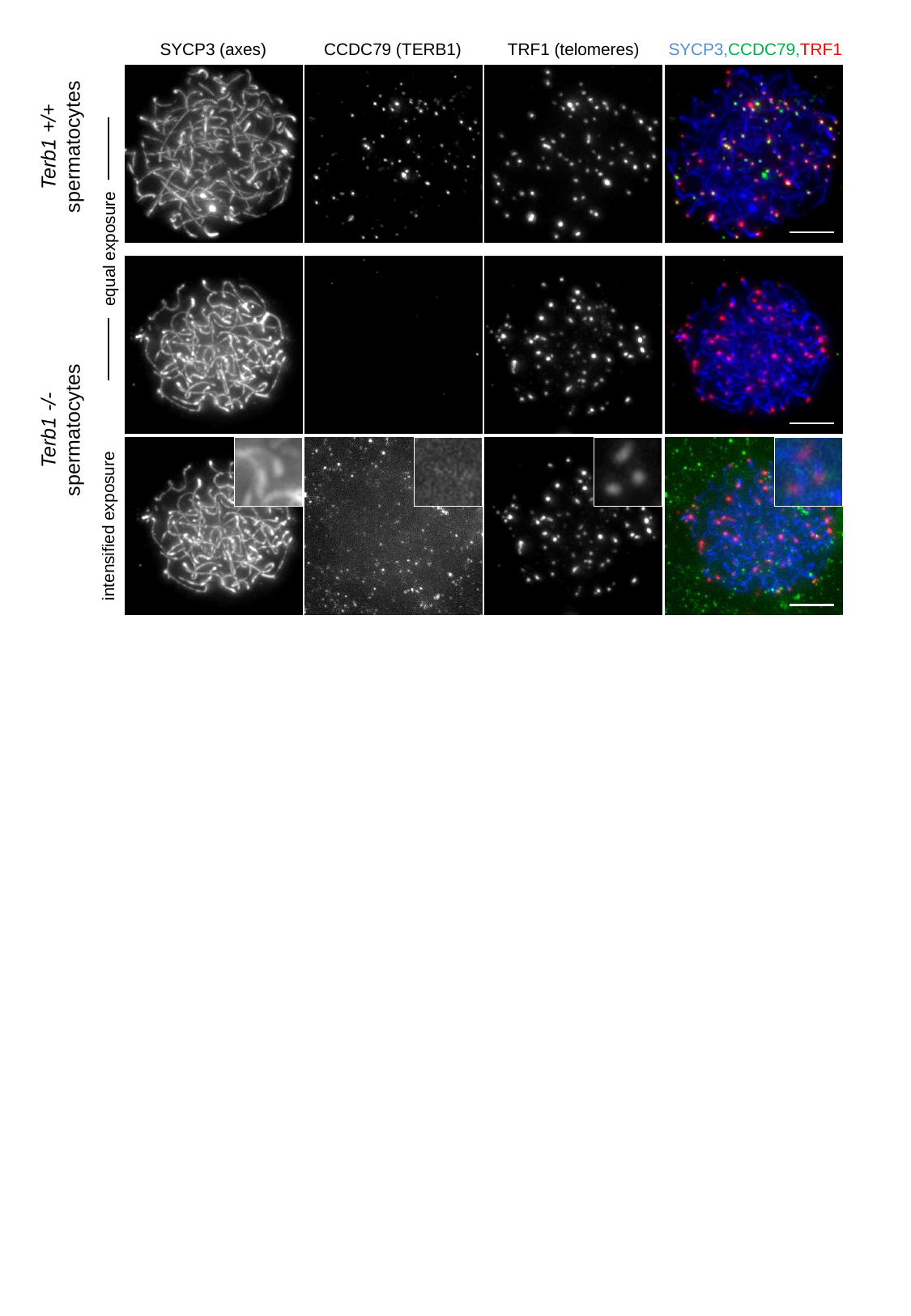

SYCP3 (axes)
CCDC79 (TERB1)
TRF1 (telomeres)
SYCP3,CCDC79,TRF1
Terb1 +/+
spermatocytes
equal exposure
Terb1 -/-
spermatocytes
intensified exposure
